# Laminin-binding protein of *Streptococcus suis* serotype 2 influences zinc acquisition and cytokine responses

**DOI:** 10.1186/s13567-022-01128-8

**Published:** 2023-01-05

**Authors:** Servane Payen, Jesús Aranda Rrodriguez, Mariela Segura, Marcelo Gottschalk

**Affiliations:** 1 Research Group On Infectious Diseases in Production Animals (GREMIP) and Swine and Poultry Infectious Diseases Research Center (CRIPA), Department of Pathology and Microbiology, Faculty of Veterinary Medicine, University of Montreal, Saint-Hyacinthe, QC J2S 2M2 Canada; 2 Department de Genètica I Microbiologia, Universitat Autónoma de Barcelona (UAB), Bellaterra (Cerdanyola del Vallès), 08193 Barcelona, Spain

**Keywords:** *Streptococcus suis*, lipoprotein, laminin-binding protein, zinc metabolism, virulence, cell adhesion and invasion, cytokines

## Abstract

*Streptococcus suis* serotype 2 is an important bacterial pathogen of swine, responsible for substantial economic losses to the swine industry worldwide. The knowledge on the pathogenesis of the infection caused by *S. suis* is still poorly known. It has been previously described that *S. suis* possesses at least one lipoprotein with double laminin and zinc (Zn)-binding properties, which was described in the literature as either laminin-binding protein (Lmb, as in the current study), lipoprotein 103, CDS 0330 or AdcAII. In the present study, the role of the Lmb in the pathogenesis of the infection caused by *S. suis* serotype 2 was dissected. Using isogenic mutants, results showed that Lmb does not play an important role in the laminin-binding activity of *S. suis*, even when clearly exposed at the bacterial surface. In addition, the presence of this lipoprotein does not influence bacterial adhesion to and invasion of porcine respiratory epithelial and brain endothelial cells and it does not increase the susceptibility of *S. suis* to phagocytosis. On the other hand, the Lmb was shown to play an important role as cytokine activator when tested in vitro with dendritic cells. Finally, this lipoprotein plays a critical role in Zn acquisition from the host environment allowing bacteria to grow in vivo. The significant lower virulence of the Lmb defective mutant may be related to a combination of a lower bacterial survival due to the incapacity to acquire Zn from their surrounding milieu and a reduced cytokine activation.

**Supplementary Information:**

The online version contains supplementary material available at 10.1186/s13567-022-01128-8.

## Introduction

*Streptococcus suis* is one of the most important swine pathogens responsible for important economic losses, causing sudden death, meningitis, and arthritis among other clinical manifestations [1]. Moreover, *S. suis* is a zoonotic agent responsible for meningitis and septic shock in humans [1]. Although the knowledge on the pathogenesis of the infection caused by this pathogen has improved in recent years, our understanding on virulence factors and the pathogenesis of the infection remains incomplete [1]. A battery of surface proteins has been proposed as being important for virulence, although most of them have only been partially confirmed [2].

Bacterial attachment to host cells by mucosal pathogens like *S. suis* is frequently associated to the first steps of the pathogenesis, leading to colonization and infection [3, 4]. Bacterial adhesion and invasion of cells would be critical to allow successful infection of the host. Indeed, bacteria have evolved with several adhesins that specifically recognize and attach to host cell surface components, including extracellular matrix proteins, such as fibronectin, collagens, and laminin [2, 3]. Several laminin-binding proteins (Lmbs) expressed on the bacterial surface have been described for different pathogenic streptococci. Lmb was first identified in *Streptococcus agalactiae* (or group B *Streptococcus*) [5], and homologs of this lipoprotein were reported in other streptococci, such as *Streptococcus pyogenes* or Group A *Streptococcus* (named as Lbp or Lsp) [6, 7] and *Streptococcus pneumoniae* (called AdcAII, see below) [8]. Most studies demonstrated that recombinant Lmbs can bind purified laminin which is a large (900 kDa), highly glycosylated multidomain protein found in all human tissues [5, 7, 9–11]. In addition, in *S*. *agalactiae*, a mutant lacking Lmb showed decreased adherence to human laminin and reduced invasion of human brain microvascular endothelial cells [12]. Lsp-negative *S*. *pyogenes* mutants were shown to be defective in adhesion and invasion of epithelial cells in vitro and they were also highly attenuated in a murine subcutaneous ulcer model [7, 13].

Although many studies mostly focused on its function as an adhesin, Lmbs (or homologous proteins) may be also involved in zinc (Zn) uptake. Indeed, crystal structure analysis of Lmb homologs from different streptococci revealed that the two globular domains form a pocket to facilitate a tetrahedral geometry for Zn-binding [8, 11, 14]. Zinc is crucial in any normal metabolic condition, but during infection this element becomes particularly critical for bacterial pathogens [15, 16]. Indeed, both host and pathogen compete for the same essential metals, which are at very low concentration in infection sites [17]. In several pathogenic streptococci, deletion of one or more Zn-acquisition lipoproteins results in lower growth under Zn-restricted conditions as well as decreased virulence, adhesion, and biofilm formation, underlining the importance of zinc metabolism during colonization and infection.

In *S. suis*, an homologue of Lmb has been found in different strains [10] and a Lmb defective mutant was shown to be avirulent in a mouse model of infection [18]. In addition, lipoproteins involved in Zn-acquisition have also been described and reported to be crucial for *S. suis* virulence [19]. However, it seems to be some confusion in the literature concerning the proteins involved in both laminin adhesion and Zn uptake. Originally, a critical role as virulence factor of the so-called Zn-binding lipoprotein 103 was reported, without any mention of a possible role as a laminin-binding protein [18]. Later, Zhang et al. described a laminin-binding protein of *S. suis* (called CDS 0330) [10], without mentioning that this protein is the same Zn-binding lipoprotein protein 103 previously described by Aranda et al*.* [18]. Interestingly, both research groups independently reported this protein as being protective using a mouse model of infection [10, 18]. More recently, Zheng et al. reported that *S. suis* possesses two Zn-binding proteins, AdcA and AdcAII. Indeed, AdcAII corresponds to both lipoprotein 103 and the CDS 0330 previously described [10, 18, 19]; yet again, no mention of a potential laminin-binding ability of such protein was discussed [19].

In the present study, we further dissected the role of the Lmb/AdcAII/lipoprotein 103/CDS 0330 as both, laminin-binding and Zn-uptake protein in the pathogenesis of the infection caused by *S. suis* serotype 2.

## Materials and methods

### Bacterial strains and growth conditions

The strains and plasmids used in this study are listed in Table 1. The classical *S. suis* serotype 2 virulent European reference P1/7 strain (wild type) was used throughout this study including for construction of the isogenic deficient mutants [18]. *S. suis* strains were cultured in Todd Hewitt broth (THB; Becton Dickinson, Mississauga, ON, Canada) as previously described [20]. *Escherichia coli* strains, and different plasmids used in this study are also listed in Table 1. For in vitro cell culture assays, bacteria were prepared as previously described [21, 22] and resuspended in cell culture medium. When needed, antibiotics (Sigma-Aldrich, Oakville, ON, Canada) were added to the media at the following concentrations: for *S. suis*, spectinomycin (Spc) at 100 μg/mL; for *E. coli*, kanamycin (Km) and spectinomycin at 50 μg/mL and ampicillin (Ap) at 100 μg/mL.

**Table 1:** **List of strains and plasmids used in this study**

Strain or plasmid	Characteristics	References
*Streptococcus suis*		
P1/7	Virulent serotype 2 ST1 strain isolated from a case of pig meningitis in the United Kingdom	[23]
5P1/7*Δlmb*	Isogenic mutant derived from P1/7; in frame deletion of *lmb* gene	[18]
P1/7 *Δlmb/ΔcpsG*	Isogenic mutant derived from P1/7; in frame deletion of *lmb* and *cpsG* gene	This study
P1/7 *ΔcpsF*	Isogenic mutant derived from P1/7; in frame deletion of *cpsF*	[21]
P1/7 comp *Δlmb*	Mutant *Δlmb* complemented with pMX1-*lmb* complementation vector	This study
*Escherichia coli*		
TOP10	F^−^ *mrcA Δ*(*mrr-hsd*RMS-*mcr*BC) φ80 *lacZΔ*M15 *Δlac*X74 *rec*A1 *ara*D139 *Δ*(*araleu*) 7697 *gal*U *gal*K *rps*L (Str^R^) *end*A1 *nup*G	Invitrogen
MC1061	Host for pMX1 derivatives	[24]
Plasmids		
pCR2.1	Ap^r^, Km^r^, pUC *ori*, *lac*Z*Δ*M15	Invitrogen
pSET4s	Spc^r^, pUC *ori*, thermosensitive pG + host3 *ori*, *lac*Z*Δ*M15	[25]
pMX1	Replication functions of pSSU1, MCS pUC19 *lac*Z SpR, *mal*X promoter of *S. suis*, derivative of pSET2	[25, 26]
p4*ΔcpsG*	pSET-4 s carrying the construct for *lmb* allelic replacement	This study
pMX1-*lmb*	pMX1 carrying intact *lmb* gene	This study

### DNA manipulations

Genomic DNA was extracted from the *S. suis* wild-type strain using InstaGene Matrix solution (BioRad Laboratories, Hercules, CA, USA). Mini preparations of recombinant plasmids were carried out using the QIAprep Spin Miniprep Kit (Qiagen, Valencia, CA, USA). Restriction enzymes and DNA-modifying enzymes (Fisher Scientific, Ottawa, ON, Canada) were used according to the manufacturer’s recommendations. Oligonucleotide primers (Table 2) were obtained from Integrated DNA Technologies (Coralville, IA, USA) and PCRs carried out with the iProof proofreading DNA polymerase (BioRad Laboratories, Mississauga, ON, Canada) or the Taq DNA polymerase (Qiagen). Amplification products were purified using the QIAquick PCR Purification Kit (Qiagen) and sequenced using an ABI 310 Automated DNA Sequencer and ABI PRISM Dye Terminator Cycle Sequencing Kit (Applied Biosystems, Carlsbad, CA, USA).

**Table 2:** **List of oligonucleotide primers used in this study**

Name	Sequence (5′ – 3′)	Construct
*cpsG*-ID1	CCAGCAAAGTATGGTGGTTTCG	p4*ΔcpsG*
*cpsG*-ID2	CCACGCCAGATTCAATGAGC	p4*ΔcpsG*
*cpsG*-ID3	GGGTTCGATAAAGATAAGCG	p4*ΔcpsG*
*cspG*-ID4	GCGAATTTGGAGTTACGAAAGC	p4*ΔcpsG*
*cpsG*-ID5	CGATTCAAATCCACGGAAAC	p4*ΔcpsG*
*cpsG*-ID6	GCTCTTGGCTAATAGCTCG	p4*ΔcpsG*
pMX1-*lmb*-F	CCGCCATGGACAGATGGGGTTTGATGCAAC	pMX1-*lmb*
pMX1-*lmb*-R	CGCGAATTCGGACAAGGCAATAATCAAGAC	pMX1-*lmb*

### Construction of the laminin-binding defective/non-encapsulated double mutant

Precise in-frame deletion of *cpsG* gene from strain P1/7 *Δlmb* [18] was constructed using splicing-by-overlap-extension PCRs as previously described [27, 28]. Overlapping PCR product were cloned into pCR2.1 (Invitrogen, Burlington, ON, Canada), extracted with EcoRI, recloned into the thermosensitive *E. coli*–*S. suis* shuttle plasmid pSET4s, and digested with the same enzyme, giving rise to the knockout vector p4*ΔcpsG*. Electroporation of wild-type strain P1/7 procedures to obtain the mutant were previously described [25]. Allelic replacement was confirmed by PCR and DNA sequencing analyses. Amplification products were purified with the QIAgen PCR Purification Kit (Qiagen) and sequenced as described above.

### Complementation of the laminin-binding defective mutant

The pMX1 vector was used for the generation of recombinant plasmids for complementation analysis (Table 1). This vector is a derivative of the *E. coli- S. suis* shuttle cloning vector pSET2 [29] and possesses the *S. suis malX* promoter for transgene expression in *S. suis*. The entire *lmb* gene was amplified from genomic DNA of *S. suis* P1/7 strain and cloned into pMX1 via EcoRI and NcoI sites, generating complementation vector pMX1-*lmb*. This plasmid was introduced into *E. coli* MC1061 for verification of the sequence and then into the deletion mutant derived from *S. suis* P1/7 to construct *lmb*-complemented mutants.

### Bacterial surface hydrophobicity assay

Relative surface hydrophobicity of the *S. suis* wild-type, Lmb and Lmb/non-encapsulated mutant strains was determined by measuring adsorption to *n*-hexadecane as previously described [30].

### Microtiter plate laminin-binding assay

Laminin binding assay was performed as previously described [31]. Briefly, Maxisorp™ flat-bottom microtiter 96-well plates (Nunc, VWR, Mississauga, ON, Canada) were coated with 5 μg/mL of laminin from human placenta from Sigma Chemical Co. (St. Louis, MO, USA) in 0.1 M carbonate coating buffer (pH 9.6) and were incubated overnight at 4 °C. The plates were washed with PBS containing 0.05% (v/v) Tween 20 (PBST, pH 7.3), and 200 μL of 3% (w/v) non-fat dry milk in PBST was added to each well to prevent non-specific bacterial binding. After 1 h at 37 °C, the wells were washed with PBST. Formaldehyde-killed bacterial suspensions of wild-type or mutant strains were added (concentration equivalent to 10^8^ CFU/mL), and the plates were incubated for 2 h at 37 °C. All unbound bacteria were subsequently removed by washing the wells with PBST. *S. suis* serotype 2-specific rabbit antiserum diluted 1/5000 in PBST prepared as previously described [31] was added to each well. This antiserum equally recognized both encapsulated and non-encapsulated serotype 2 *S. suis* as previously shown [31]. The plates were incubated for 1 h at 37 °C and wells were then washed, followed by the addition of horseradish peroxidase-labelled anti-rabbit IgG (Jackson Immunoresearch Laboratories Inc., West Grove, PA, USA) (diluted 1/8000 in PBST). The plates were incubated for 1 h at 37 °C with the secondary antibody. After washing, 3,3′,5,5′-tetramethylbenzidine (Zymed, San Francisco, CA, USA) was used as the enzyme substrate according to the manufacturer’s instructions. The reactions were stopped by adding 25 μL of H_2_SO_4_ (1 N) and were read at 450 nm. Uncoated wells served as background controls. Casein-coated wells served as a control for non-specific adhesion of *S. suis* to protein-coated wells. Each experiment was repeated at least 3 times.

### Bacterial adhesion and invasion assays using porcine tracheal epithelial and brain microvascular endothelial cells

The neonatal porcine tracheal epithelial cell line (NPTr) and the porcine brain microvascular endothelial cell line (PBMEC) were used and cultured until confluence as previously described [32, 33]. Cells were infected with 1 × 10^6^ CFU/well (multiplicity of infection (MOI) = 10) of the different *S. suis* strains and incubated for 2 h at 37 °C in 5% CO_2_. The adhesion assay, which quantifies total cell-associated bacteria (surface-adherent and intracellular bacteria), and invasion assay (using the antibiotic protection assay) were performed as previously described [32, 33].

### *S. suis* virulence mouse model of systemic infection

A C57BL/6 J mouse model of infection was used. These studies were carried out in strict accordance with the recommendations of and approved by the University of Montreal Animal Welfare Committee guidelines and policies, including euthanasia to minimize animal suffering by the use of humane endpoints, applied throughout this study when animals were seriously affected (mortality was not an endpoint measurement). Thirty 6-week-old female C57BL/6 J (Jackson Research Laboratories, Bar Harbor, ME, USA) were used for these experiments (15 mice per group). Early stationary phase bacteria were washed twice in phosphate-buffered saline, pH 7.4, and resuspended in THB [34–36]. Bacterial cultures were appropriately diluted and plated on THB agar (THA) to accurately determine bacterial concentrations. Mice were inoculated with 1 × 10^7^ CFU via the intraperitoneal route and health and behavior monitored at least thrice daily until 72 h post-infection and twice thereafter until the end of the experiment (12 days post-infection) for the development of clinical signs of sepsis, such as depression, swollen eyes, rough hair coat, prostration, and lethargy. For bacteremia studies, blood samples were collected from the caudal vein of surviving mice 12 h, 24 h and 48 h post-infection and plated as previously described [36].

### Measurement of plasma (systemic) pro-inflammatory mediators

In addition, 8 mice per group were intraperitoneally mock-infected (THB) or infected with 1 × 10^7^ CFU of wild-type or *Δlmb* mutant strains and blood samples were collected 12 h post-infection by intracardiac puncture following euthanasia and anti-coagulated with EDTA (Sigma-Aldrich) as previously described [36, 37]. Plasma supernatants were collected following centrifugation at 10 000 × *g* for 10 min at 4 °C and stored at −80 °C. The 12 h post-infection time point was selected to obtain maximal pro-inflammatory mediator production in the absence of significant mouse mortality as determined previously [36]. Plasmatic concentrations of interleukin (IL)-6 and C-X-C motif chemokine ligand (CXCL) 1 were quantified by sandwich. ELISA using pair-matched antibodies from R&D Systems (Minneapolis, MN, USA) according to the manufacturer’s recommendations.

### Phagocytosis assay

J774A.1 murine macrophages (ATCC TIB-67; Rockville, MD, USA) were maintained in Dulbecco’s Modified Eagle’s Medium (Gibco, Burlington, ON, Canada) supplemented with 10% fetal bovine serum (Gibco) and grown at 37 °C with 5% CO_2_. Confluent cell cultures were scraped, seeded at 1 × 10^5^ cells/mL, and incubated for 3 h at 37 °C with 5% CO_2_ to allow cell adhesion. Cells were infected by adding 1 × 10^7^ CFU/mL of bacterial suspension in complete culture medium (MOI = 100), incubated for 2 h at 37 °C with 5% CO_2_, and phagocytosis assays performed as previously described using the antibiotic protection assay [21].

### *S. suis* activation of marrow-derived dendritic cells (bmDC)

The femur and tibia from C57BL/6 J mice (Jackson Research Laboratories) were used to generate bmDCs, as previously described [21]. Briefly, hematopoietic bone marrow stem cells were cultured in complete culture medium (RPMI-1640 supplemented with 5% heat-inactivated fetal bovine serum, 10 mM HEPES, 2 mM L-glutamine, and 50 µM 2-mercaptoethanol (Gibco, Burlington, ON, Canada) and complemented with 20% granulocyte-macrophages colony-stimulating factor from mouse-transfected Ag8653 cells [38]. Cell purity was confirmed to be at least 90% CD11c + by flow cytometry as previously described [21]. Albeit this culture system cannot completely rule out the presence of other innate cells such as macrophages, it represents an enriched source of bmDCs [39].

All experiments were performed in the absence of endotoxin (lipopolysaccharide) contamination and under non-toxic conditions (data not shown), the latter being evaluated by the lactate dehydrogenase release with the CytoTox 96^®^ Non-Radioactive Cytotoxicity Assay (Promega, Madison, WI, USA). Since the P1/7*Δlmb* mutant strain did not grow in the RPMI-1640 complete medium, heat-killed *S. suis* suspensions (prepared as previously described [40]) of all tested strains were used for bmDC stimulation at a concentration equivalent to 2 × 10^9^ CFU/mL. Cells were resuspended at 1 × 10^6^ cells/mL in complete medium and stimulated with the different heat-killed *S. suis* strains. Supernatants were collected at 4 h, 6 h, 8 h, 12 h and 16 h following stimulation with heat-kill *S. suis*, incubation times at which secreted cytokine levels were maximal in the absence of cytotoxicity (data not shown) [20, 21]. Mock-infected cells served as negative controls. Secreted levels of tumor necrosis factor (TNF), interleukin (IL)-6, C-C motif chemokine ligand (CCL) 3, and C-X-C motif chemokine ligand (CXCL) 1 were quantified by sandwich ELISA using pair-matched antibodies from R&D Systems (Minneapolis, MN, USA) according to the manufacturer’s recommendations.

### Bacterial growth analysis

Bacterial growth experiments were performed in microtubes (500-μL culture volume). A bacterial overnight culture grown (1 × 10^4^ CFU/mL) in THB was used to inoculate THB, plasma naturally poor in zinc, plasma with various ZnSO_4_ concentrations (from 0 to 50 μM) and plasma with ZnSO_4_ and the addition of the appropriate concentration of the chelating agent Tetrakis-(2-Pyridylmethyl) ethylenediamine (TPEN, Sigma Aldrich). Growth was followed during 24 h of incubation at 37 °C. The total numbers of CFU/mL were evaluated at different incubation times.

### Statistical analyses

Normality of data was verified using the Shapiro–Wilk test. Accordingly, parametric (unpaired t-test) or non-parametric tests (Mann-Whitney rank sum test), where appropriate, were performed to evaluate statistical differences between groups. Log-rank test was used to compare survival rates between wild-type-infected mice and those infected with mutant strains. Each in vitro test was repeated in at least three independent experiments. *p* < 0.05 was considered as statistically significant.

## Results

### Surface hydrophobicity of the strains included in this study

Previous studies have demonstrated that deletion of either *cpsG* or *cpsF* biosynthesis genes from *S. suis* results in a non-encapsulated phenotype [30, 41]. An isogenic mutant in which the *cpsG* gene, encoding a glycosyltransferase, was deleted from the P1/7 *Δlmb* strain was constructed and compared to the wild-type P1/7, P1/7 *Δlmb* and P1/7 *ΔcpsF.* Surface hydrophobicity of P1/7 *Δlmb* was low (less than 6%) and comparable to that of wild-type P1/7 (Additional file 1). Meanwhile, and as expected, deletion of the *cpsG* gene significantly increased surface hydrophobicity, with similar values to those obtained with P1/7 *ΔcpsF* non-encapsulated mutant (Additional file 1). These results indicate that the Lmb does not play any role in the surface hydrophobicity of the strain tested.

### Lack of lipoprotein Lmb does not affect *S. suis*-binding to laminin

**Figure 1:**
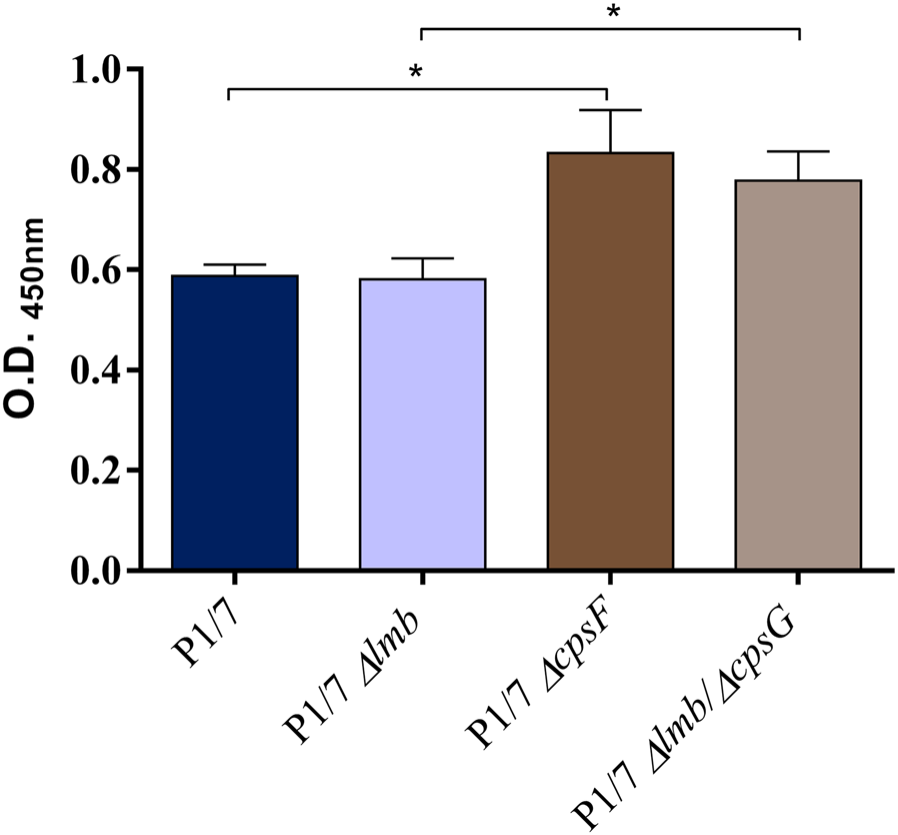
**The *Δlmb* mutant is not impaired in its capacity of adhesion to laminin.** Adhesion to laminin of the wild-ype P1/7 strain (dark blue), *Δlmb* (light blue), *ΔcpsF* (dark brown) and *Δlmb/ΔcpsG* (light brown) mutant strains as evaluated by ELISA. Data represent the optical density (O.D.)_450_ mean ± SEM from at least three independent experiments. *Indicates a significant difference (*p* < 0.05).

Although previous studies revealed that purified Lmb of *S. suis* could bind human laminin [31], the ability of a *Δlmb* mutant to bind immobilized human placental laminin was not previously evaluated. Binding capacity to laminin of the *Δlmb* mutant was comparable to that of the wild-type strain (Figure 1). It was also confirmed that a non-encapsulated mutant strain is able to bind laminin more efficiently than the wild-type strain, as previously described [31]. Interestingly, adhesion to laminin of a double mutant *Δlmb*/non-encapsulated (*Δlmb*/*ΔcpsG*) was similar to that observed with the single non-encapsulated *ΔcpsF* mutant (Figure 1). Taken, together, these results show that Lmb would not be critical for the interaction between *S. suis* and laminin, even in the absence of the capsular polysaccharide (CPS).

### Lack of lipoprotein Lmb does not impair the adhesion to and invasion of epithelial and endothelial swine cells

**Figure 2:**
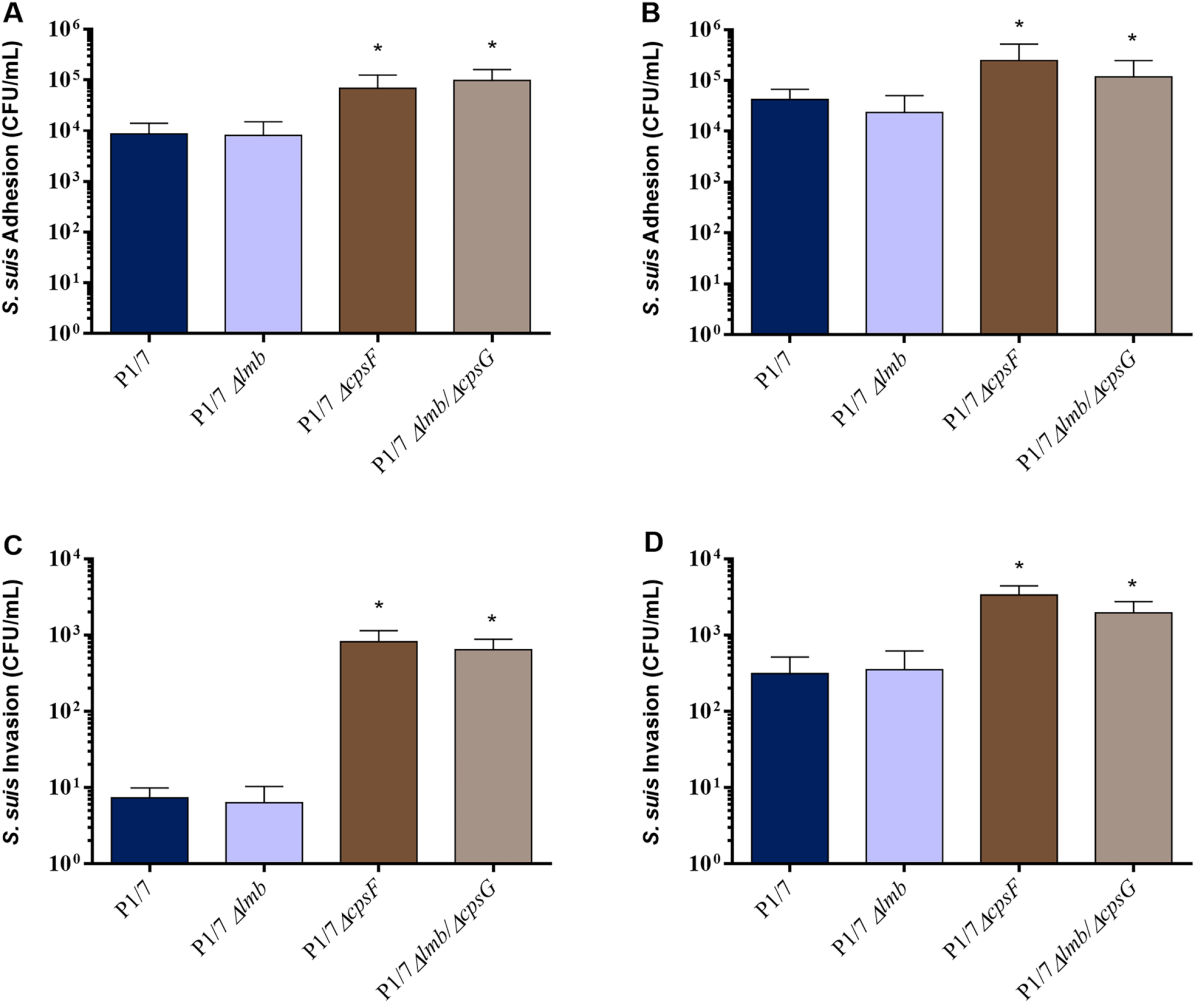
**The *Δlmb* mutant is not impaired in its capacity of adhesion to and invasion of epithelial and endothelial cells.** Adhesion to and invasion of NPTr (**A**, **C**) and PBMEC (**B**, **D**) of wild-type P1/7 strain (dark blue), *Δlmb* (light blue), *ΔcpsF* (dark brown) and *Δlmb/ΔcpsG* (light brown) mutant strains after 2 h of incubation. *Indicates a significant difference (*p* < 0.05). Each bar represents the mean bacterial concentration (CFU/mL)+SEM from at least three independent experiments.

The role of the Lmb protein in the adhesion/invasion to NPTr and PBMEC cells was evaluated using the generated mutant strains. For both cell types, and as expected [42, 43], the non-encapsulated mutant strain significantly adhered and invaded cells more efficiently than the wild-type strain (Figures 2A–D). However, the *Δlmb* mutant similarly adhered and (weakly) invaded NPTr (Figures 2A, B) and PBMEC (Figures 2C, D) cells. Moreover, the *Δlmb/ΔcpsG* double mutant adhered and invaded the two cell types similarly to the non-encapsulated *ΔcpsF* mutant. These results indicate that the Lmb does not play a critical role in the adhesion/invasion of the epithelial and endothelial cells tested, even in the absence of the CPS.

### Presence of the lipoprotein Lmb is required for full virulence of *S. suis*

**Figure 3:**
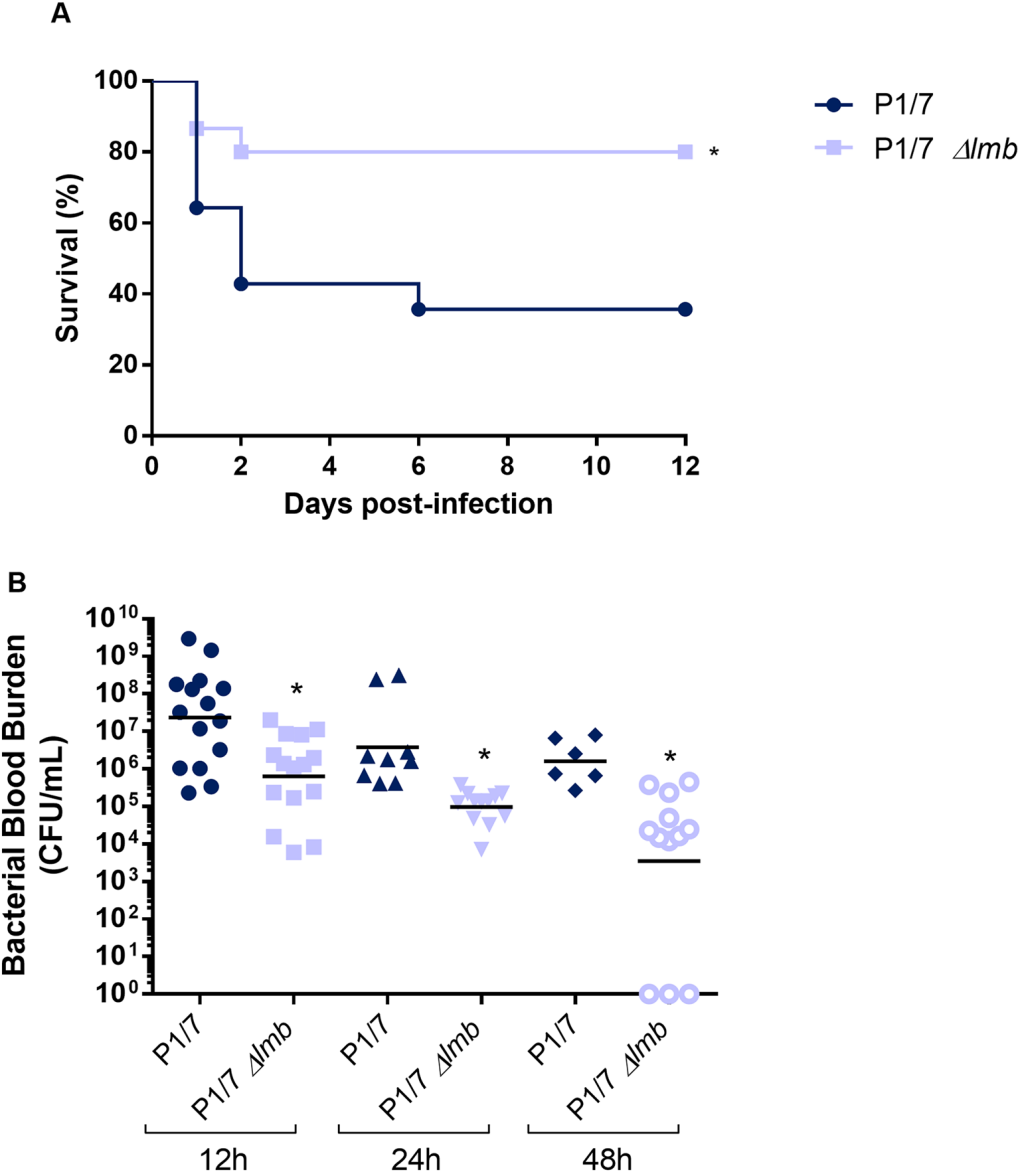
**Presence of the lipoprotein Lmb is important for *S. suis* systemic virulence and blood persistence following intraperitoneal inoculation.** Survival (**A**) and blood bacterial burden at 12, 24 and 48 h post-infection (**B**) of C57BL/6 mice following intraperitoneal inoculation of the *S. suis* virulent wild-type P1/7 strain (dark blue) and *Δlmb* mutant strain (light blue). Data represent survival curves (**A**) (*n* = 15) or geometric mean (**B**) (*n* = survived mice at each time point). *(*p* < 0.05) indicates a significant difference between survival or blood bacterial burden of mice infected either with the wild-type or the *Δlmb* mutant strain.

To confirm the role of Lmb in *S. suis* virulence and development of clinical disease, a well-characterized C56BL/6 mouse model of infection was used [36, 44]. Wild-type strain-infected mice rapidly developed clinical signs of systemic disease characteristic of septic shock with 60% of mice succumbing to infection within 48 h (Figure 3A). On the other hand, only 20% of mice infected with the *Δlmb* mutant succumbed to disease (Figure 3A). These mice developed transient signs of infection such as rough coat hair following inoculation of bacteria and rapidly recovered and presented a normal behavior. These results confirm that the Lmb lipoprotein is crucial for the virulence of *S. suis* serotype 2 strain P1/7.

Blood bacterial burden was also evaluated at the early infection times of 12 h, 24 h and 48 h (Figure 3B). Twelve hours following infection, mice infected with the wild-type strain presented elevated blood bacterial burdens averaging 1 × 10^7^ CFU/mL (Figure 3B). Moreover, infection with the wild-type strain resulted in elevated bacterial burdens after 24 h and 48 h infection. On the other hand, mice infected with the *Δlmb* mutant show a significant decrease in blood bacterial burden at 12, 24 h and 48 h post-infection. This difference would have been more evident at 48 h after infection considering that almost 60% of mice infected with the wild-type P1/7 strain were already dead and the viable counts were performed with survived (less affected) mice only. These data indicate that the *Δlmb* mutant strain presents less survival capacity within the bloodstream of infected mice.

**Figure 4:**
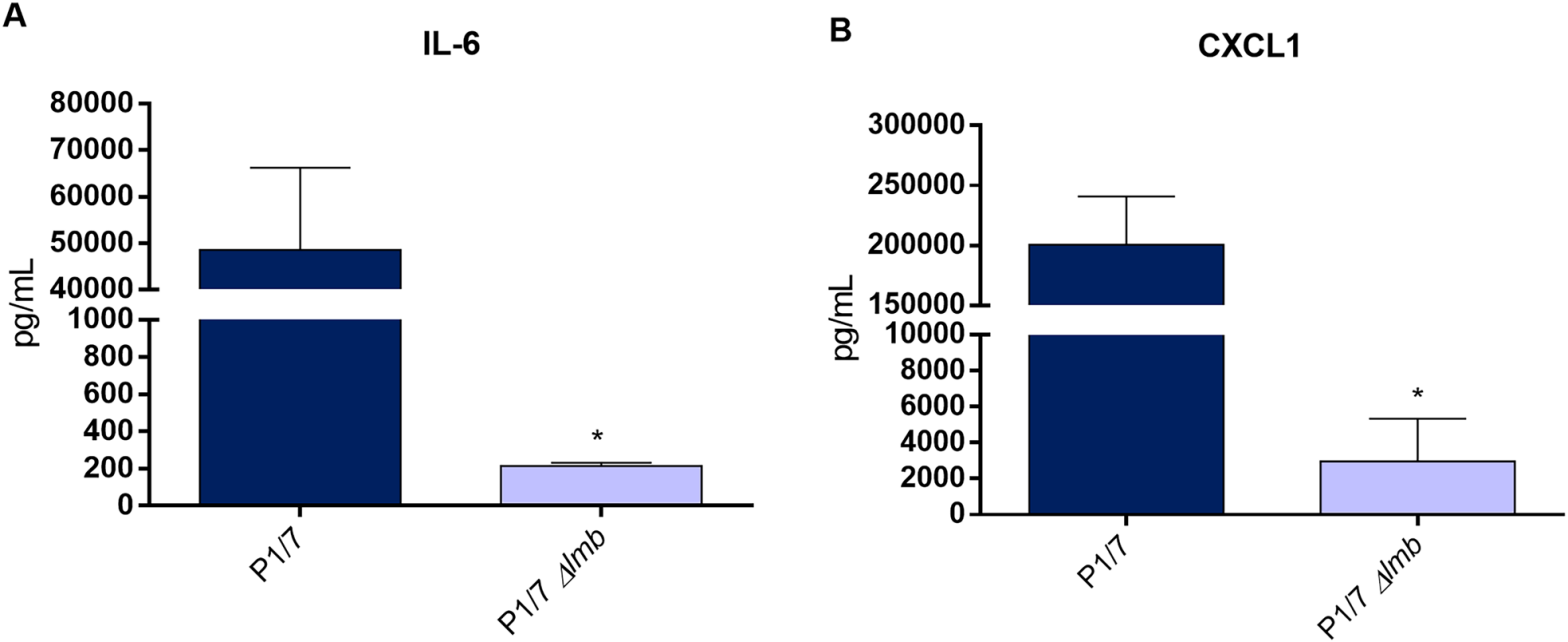
**Absence of the lipoprotein Lmb significantly decreases the cytokine levels in mouse plasma from infected animals.** Plasma levels of IL-6 (A) and CXCL1 (B) in mice 12 h following intraperitoneal inoculation of the *S. suis* serotype 2 wild-type strain P1/7 (dark blue) or *Δlmb* mutant strain (light blue). Data represent mean ± SEM (*n* = 8). *p < 0.05 indicates a significant difference between plasma levels of mice infected with the mutant strain when compared to the wild-type P1/7 strain.

In addition, the inflammatory response of animals infected with the P1/7 and *Δlmb* mutant strain was evaluated. IL-6 and CXCL1 concentration were measured after 12 h of infection. The concentrations of these plasma mediators were significantly lower for the *Δlmb* mutant strain when compared to P1/7 wild-type strain (Figure 4).

### Lmb does not promote *S. suis* resistance to phagocytosis by murine macrophages

**Figure 5:**
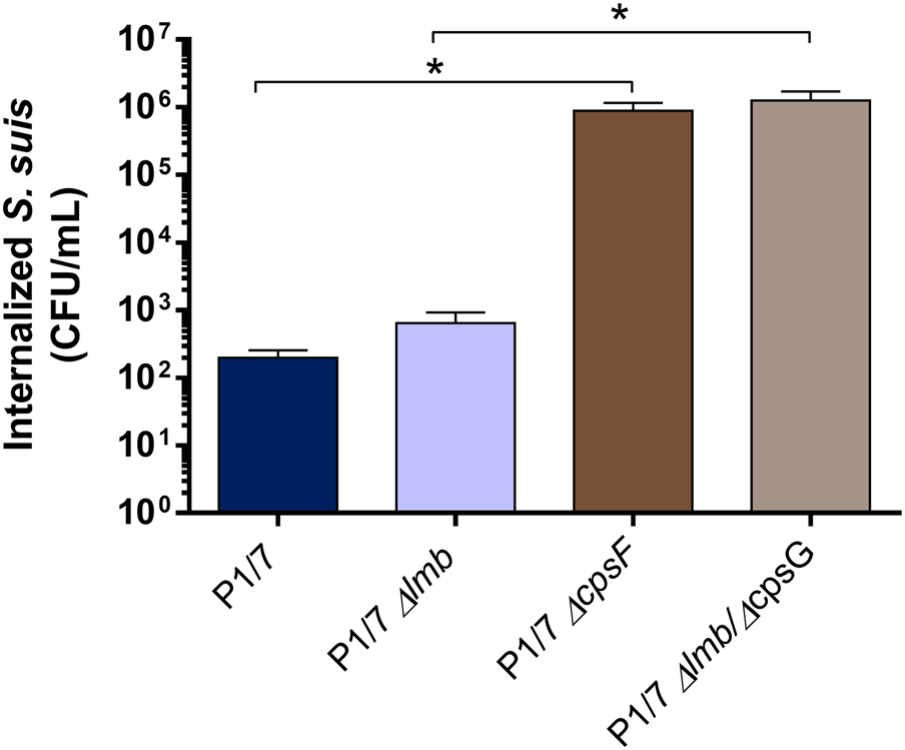
**The lipoprotein Lmb is not involved in *S. suis* phagocytosis resistance to J774A.1 murine macrophages.** Internalization of the P1/7 wild-type strain (dark blue), *Δlmb* (light blue), *ΔcpsF* (dark brown) and *Δlmb/ΔcpsG* (light brown) mutant strains after 2 h of incubation. * (*p* < 0.05) indicate a significant difference between the wild-type and *ΔcpsF* mutant and a significant difference between the *Δlmb* and *Δlmb/ΔcpsG* mutants. Each bar represents the mean bacterial concentration (CFU/mL) + SEM from at least three independent experiments.

One of the hypotheses that may explain that the *Δlmb* mutant survives less in the bloodstream of infected mice is an increased susceptibility to phagocytosis. In vitro phagocytosis studies with murine macrophages showed that the wild-type strain was less phagocytosed than the non-encapsulated *ΔcpsF* mutant, as previously described [30] (Figure 5). The *Δlmb* mutant was internalized similarly to the wild-type P1/7 strain, whereas the *Δlmb/ΔcpsG* double mutant was phagocytosed similarly to the non-encapsulated *ΔcpsF* mutant. These results indicate that the lipoprotein Lmb does not play any role in the resistance to the phagocytosis.

### The lipoprotein Lmb partially regulates bmDC release of cytokines

**Figure 6:**
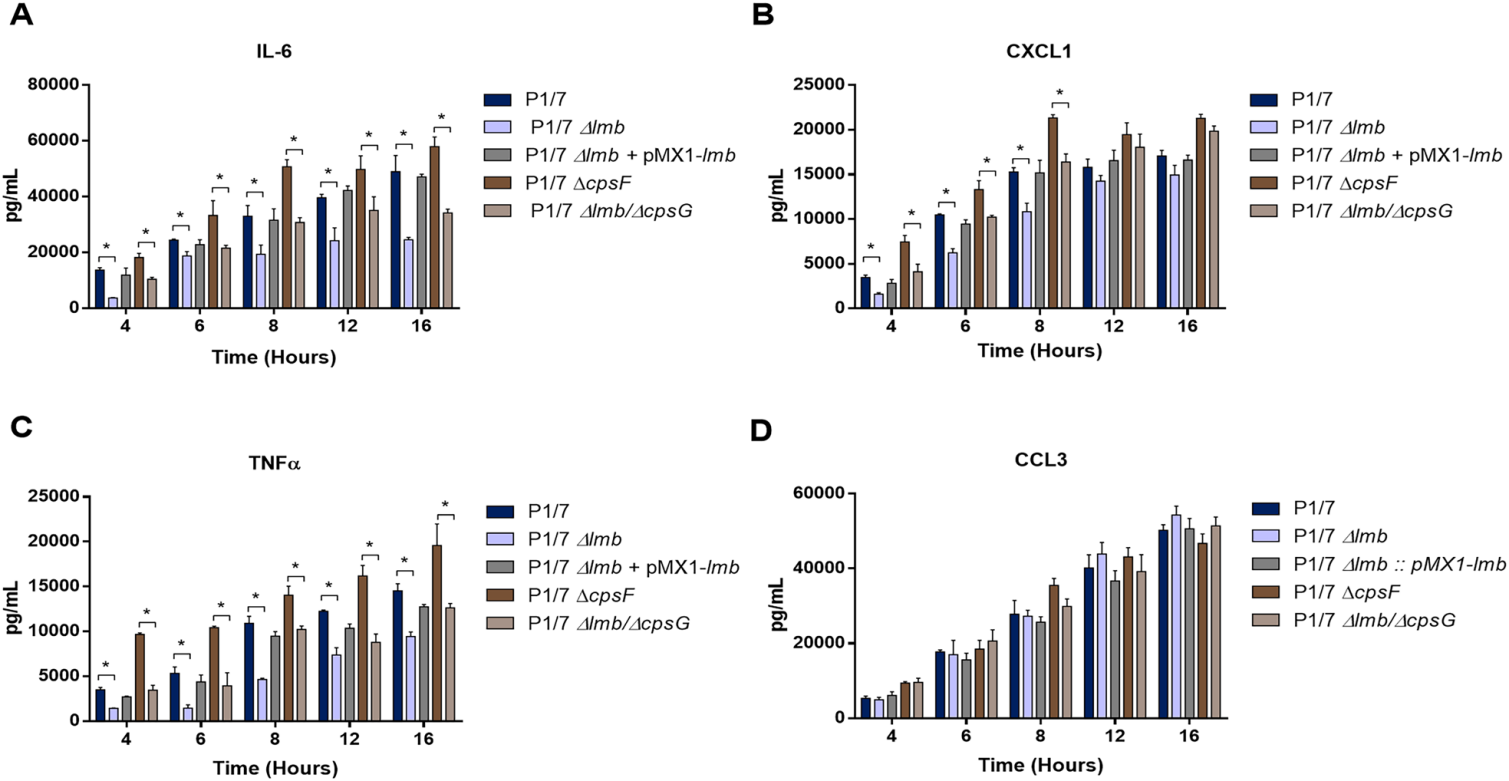
**Presence of the lipoprotein Lmb modulates *S. suis* induced dendritic cell (DC) cytokine production.** Pro-inflammatory cytokine production by bmDCs following activation with heat killed bacteria of the wild-type P1/7 strain (dark blue), *Δlmb* (light blue), *Δlmb*:: pMX1-*lmb* (grey), *ΔcpsF* (dark brown) and *Δlmb/ΔcpsG* (light brown) mutant strains. Production of IL-6 (**A**), CXCL1 (**B**), TNF (**C**) and CCL3 (**D**). Data represent the mean+SEM of at least three independent experiments. *Indicates a significant difference (*p* < 0.05).

The role of the Lmb lipoprotein in cytokine release was evaluated using bmDCs as an innate immune cell model, given that dendritic cells play a critical role during *S. suis* pathogenesis and that their inflammatory response to *S. suis* has been well-characterized [20, 21]. bmDCs were activated for up to 16 h with heat-killed bacteria. For all experiments and at all incubation times, control mock infected cells presented negligible cytokine values < 300 pg/mL (not shown). The *Δlmb* and *Δlmb/cpsG* mutant strains induced significant lower levels of IL-6 and TNF (Figures 6A, C) and, to a lesser extent, CXCL1 (Figure 6B), when compared to wild-type P1/7 and non-encapsulated *ΔcpsF* mutant strains, respectively (Figure 6). The complemented *Δlmb:*: pMX1-*lmb* strain restored the capacity of cytokine induction of the *Δlmb* mutant to values similar to those of the wild-type strain (Figure 6). On the other hand, the presence of Lmb did not affect the induction of CCL3 (Figure 6D). These results indicate that the Lmb lipoprotein is one of the *S. suis* components responsible for cytokine release.

### The lipoprotein Lmb is essential for *S. suis* growth under zinc starvation conditions

**Figure 7:**
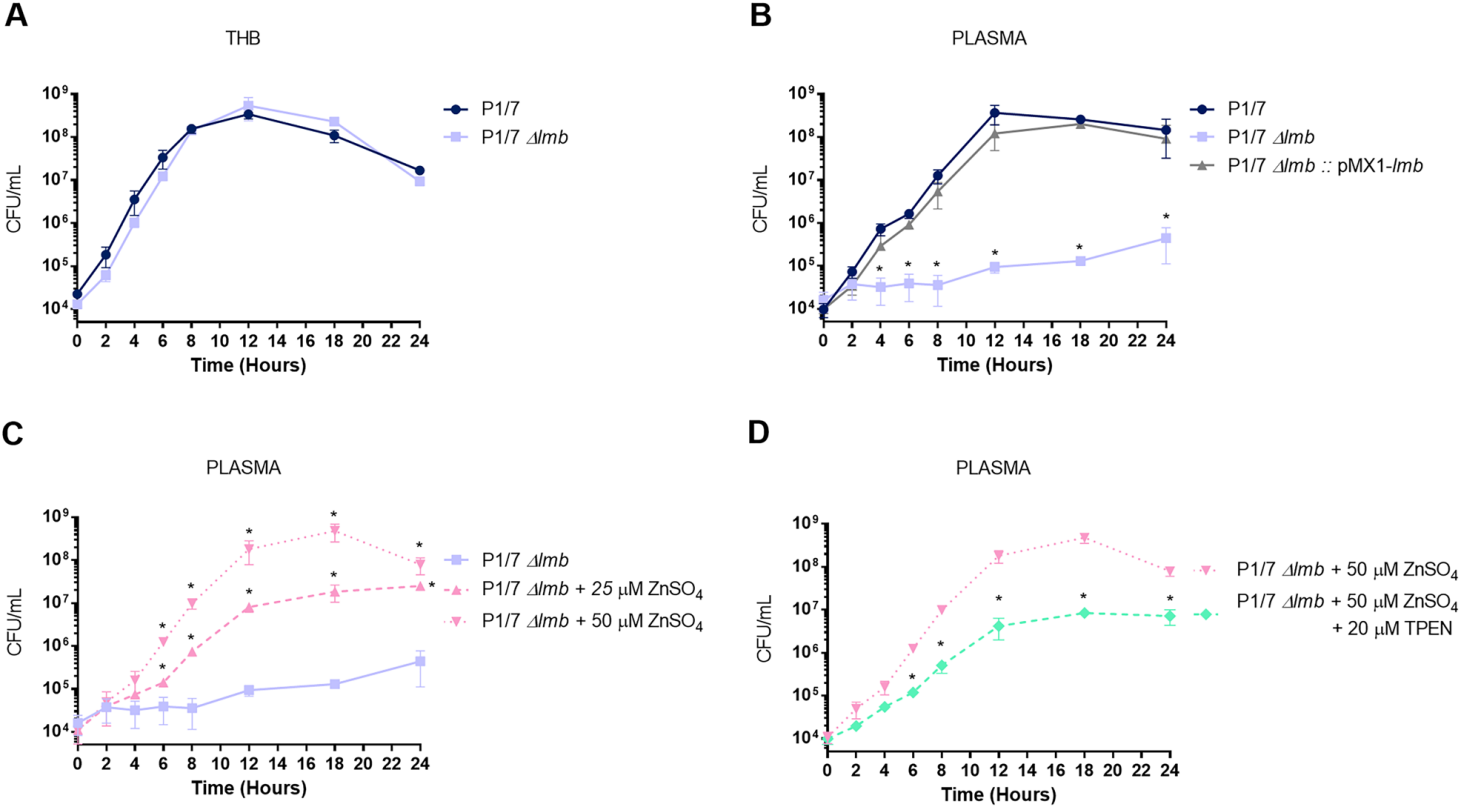
**Growth of Δlmb mutant strain under different conditions.** Growth of the wild-type P1/7 strain (dark blue), *Δlmb* (light blue) and *Δlmb*::pMX1-lmb (grey, plasma only) in THB (A), plasma (B), plasma supplemented with 25 µM ZnSO4 (pink dash) or 50 µM ZnSO4 (pink dot) (C) and plasma supplemented with 50 µM ZnSO4 (pink dot) added with 20 µM TPEN (green). *(*p* < 0.05) indicate a significant difference between the wild-type and *Δlmb* mutant in plasma, between *Δlmb* mutant with and without supplementation of ZnSO4 in plasma or between *Δlmb* mutant with supplementation of ZnSO4 and with supplementation of ZnSO4 with addition of TPEN. Each point represents mean bacterial concentration (CFU/mL) + / − SEM of at least three different independent experiments.

Considering the limited role observed for the Lmb as laminin-binding protein, the second characteristic already described in the literature for this lipoprotein (Zn uptake) was evaluated. First, the wild-type P1/7 and *Δlmb* strains cultured in rich media (THB) presented similar good growth rate, as evaluated by bacterial counts (Figure 7A). In contrast, when cultured in plasma (a poor medium that mimic in vivo conditions), growth of the *Δlmb* mutant strain was seriously reduced when compared to the wild-type strain (Figure 7B). The complemented *Δlmb* mutant completely restored the growth to the levels of the wild-type strain. To investigate if the loss of Lmb was associated with a reduced ability to grow under conditions of zinc starvation, ZnSO_4_ was added to the plasma at concentrations of 25 and 50 µM (Figure 7C). Growth of *Δlmb* mutant was partially and totally restored with 25 µM and 50 µM ZnSO_4_, respectively, indicating a dose-effect of zinc concentration (Figure 7C). Finally, the chelating agent N,N,N’,N’-Tetrakis-(2-pyridylmethyl)ethylenediamine (TPEN), a cell permeable high-affinity Zn^2+^ chelator used to reduce concentration of zinc in zinc homeostasis studies (15, 49, 51) was added to the plasma supplemented with ZnSO_4_ (Figure 7C) [13, 45, 46]. Addition of 20 μM TPEN in plasma supplemented with 50 µM ZnSO_4_ impaired the growth of the *Δlmb* mutant strain (Figure 7D).

## Discussion

The pathogenesis of the infection caused by *S. suis* is only partially known and the definition of critical virulence factors is controversial [2]. During the first steps of the infection, the interaction of microorganisms with extracellular matrix proteins can promote bacterial colonization and invasion [47]. It has been shown that *S. suis* is able to interact with several of these proteins, including laminin [31]. On the other hand, in order for bacterial pathogens to survive inside their hosts, they need to efficiently acquire and use the available nutrients from their surrounding medium. One of these components playing an important role in the host-pathogen interplay is zinc (Zn) [48]. Zn-binding lipoproteins have also been described in *S. suis* as being critically important for bacterial fitness and survival during the infection [19]. Interestingly, one of these *S. suis* laminin- and Zn-binding proteins is a single lipoprotein with a double function previously identified as lipoprotein 103, Lmb CDS 0330 or AdcAII [18, 19]. This protein (or genes coding for it) presents high homology with that of other streptococci [10], in which this double function has also been described [5, 7, 13, 46]. In the case of *S. suis*, it is not clear whether the laminin-binding or the Zn-binding capacities of this protein (or both) plays an important role during the infection. In the present study, the more precise role of this lipoprotein (called Lmb in the current study for simplicity purposes) in the pathogenesis of the infection has been studied.

Based on the previous observation that some *S. suis* strains are able to bind laminin [31], Zhang et al. showed that *S. suis* possesses a *lmb* gene which is homologous to those of *S. pyogenes*, *S. agalactiae* and *S. pneumoniae* and the cloned and purified protein clearly adhered to laminin in vitro. However, it is not clear if this lipoprotein is mainly responsible for the binding of *S. suis* to laminin. Results from the current study using a Lmb-defective mutant indicate that the Lmb does not play a critical role in the bacterial binding to this extracellular matrix protein. Although Lmb was reported as a surface protein [10], it was previously described that the presence of the CPS may prevent *S. suis* to attach to laminin by hindering surface proteins [31]. Hence, a double mutant without Lmb and CPS was constructed and tested. The *ΔcpsF* non-encapsulated mutant adhered significantly more to laminin when compared to the wild-type strain confirming previous results. However, the double *Δlmb*/*ΔcpsG* mutant adhered similarly to the non-encapsulated mutant, indicating that even in the absence of CPS the surface expressed Lmb does not seem to play an important role in *S. suis* adhesion to the laminin. The presence of additional proteins able to bind laminin that have been described as being present in *S. suis* serotype 2 may compensate the absence of Lmb [49, 50].

The role of the Lmb in the adhesion/invasion of host cells seems to vary among different streptococci. For example, the presence of the Lmb seems to be important for *S. agalactiae* colonization of the epithelium and subsequent translocation into the bloodstream, having a tropism for the central nervous system [51]. The Lbp from *S. pneumoniae* has also been reported to play an important role in the adhesion of the pathogen to human microvascular endothelial cells [52] and *S. pyogenes Δlmb* (or Lsp, or Lbp) mutants show a decrease adhesion only [6] or a decrease in adhesion and invasion [7] to host cells. Results of the current study showed that the Lmb does not play any major role in *S. suis* adhesion to or invasion of swine respiratory epithelial and brain microvascular endothelial cells, which correlates with the fact that this protein is not critical for laminin binding. As mentioned, other proteins playing similar overlapped functions may compensate and allow *S. suis* to adhere to this extracellular matrix protein and host cells.

There are several studies on the role of a Lmb (or homologous proteins) as Zn-binding proteins of streptococci [5–8], with the exception of that of *S. pneumoniae* which plays an important role as a Zn-binding lipoprotein but it does not bind laminin [53]. As mentioned, two Zn-binding proteins have been described in *S. suis*: AdcA and AdcAII [19]. Although both lipoproteins may play a role in Zn-binding, Zhang et al. reported that *AdcAII* expression is significantly more up-regulated than *AdcA*, but only the absence of both lipoproteins affected survival under Zn-restricted conditions and virulence in a mouse model of infection [19]. Results of the current study confirm only partially such observations. Indeed, the *Δlmb* mutant (which corresponds to the *ΔAdcAII* previously described) could not grow under Zn-restricted conditions (plasma) and the addition of Zn restored this defect. In addition, the complemented-mutant was able to grow at similar levels than the wild-type strain. In addition, and also different from the study of Zhang et al. [19], in the current work, the *Δlmb* mutant presented a markedly lower virulence than the wild-type strain, confirming previous results reported by Aranda et al. [18], who used mice from a different background (BALB/c). The critical role of *ΔAdcAII* has also been observed in *S. pneumoniae* (16), whereas both *ΔAdcA* and *ΔAdcAII* presented less virulence than a wild-type strain of Group A *Streptococcus* [54]. In our hands, the presence of *ΔAdcAII* is critical for Zn uptake and virulence in *S. suis* serotype 2. Differences observed among the *S. suis* studies might be explained by the background of the *S. suis* strains used: the current study and that of Aranda et al. [18] used the virulent P1/7 sequence type (ST) 1 as wild-type reference strain, whereas Zhang et al. [19] used a ST7 highly virulent strain from China. Previous studies have also reported some differences within *S. suis* serotype 2 strains from different background in the pathogenesis of the infection [22, 55]. It may be hypothesized that regulation of Zn may vary depending on the virulence/phenotype of the strain.

The lower virulence of the *Δlmb* mutant can be explained by a lower survival (lower bacteremia) in vivo as shown in the current study. This would be directly related to the in-vivo Zn-restricted conditions rather to a higher susceptibility of the mutant to bacterial killing. Indeed, no difference in the phagocytosis rate between the *Δlmb* mutant and the wild-type strains could be observed. In addition, induction of production of large amounts of pro-inflammatory mediators leading to exacerbated inflammation is a hallmark of *S. suis* infections and is responsible, at least in part, for host death [56]. Although the current study clearly showed a lower cytokine concentration in mice infected with the *Δlmb* mutant, this effect might be the simple consequence of lower bacteremia. However, in addition to this, in vitro results showed that the presence of the Lmb is also important as cytokine activator. Similarly, the laminin-binding protein from *S. pneumoniae* (Lbp) was reported to activate human brain microvascular-endothelial cells [52], whereas that of *S. agalactiae* was not involved in IL-8 release when using the same cells [12]. As lipoproteins have been clearly shown to be responsible for the cytokine activation of cells by *S. suis* [57, 58], it can also be hypothesized that the lower cytokine production in vivo by the *Δlmb* mutant is a double combination of lower bacteremia and reduced activation due to the absence of this lipoprotein.

In summary, results from the current study showed that Lmb does not play an important role in the laminin-binding activity of *S. suis* serotype 2. In addition, the presence of this lipoprotein does not influence bacterial adhesion to and invasion of porcine respiratory epithelial and brain endothelial cells and it does not increase the susceptibility of *S. suis* serotype 2 to phagocytosis. On the other hand, this lipoprotein was shown to play an important role as cytokine activator when tested in vitro with dendritic cells. Finally, the Lmb plays a critical role in Zn acquisition from the host environment allowing bacteria to grow in vivo. The significant lower virulence of the Lmb defective mutant may be related to a combination of a lower bacterial survival due to the incapacity to acquire and use Zn from their surrounding milieu and a reduced cytokine activation in the absence of this lipoprotein. Since the gene coding for this protein is also present in other serotypes of *S. suis* (data from GeneBank, not shown), it would be interesting to confirm if the role of the Lmb described in the current study applies also to other pathogenic serotypes of *S. suis*.

## Supplementary Information

**Additional file 1: Absence of Lmb does not influence *S. suis* surface hydrophobicity.** Surface hydrophobicity of of the wild-type P1/7 strain (dark blue), *Δlmb* (light blue), *ΔcpsF* (dark brown) and *Δlmb/ΔcpsG* (light brown) strains was determined using *n*-hexadecane. Data represent the mean ± SEM from at least three independent experiments. *Indicates a significant difference (*p* < 0.05).

## Abbreviations

Ap: ampicillin
CFU: colony-forming unit
CCL: C-C motif chemokine ligand
CXCL: C-X-C motif chemokine ligand
DC: dendritic cell
ELISA: enzyme-linked immunosorbent assay
Ig: immunoglobulin
IL: interleukin
Km: kanamycin
LB: Luria-Bertani
Lmb: laminin binding protein
Nptr: Neonatal Porcine Tracheal Epithelial cell
PBMEC: Porcine Brain Microvascular Endothelial cell
PCR: polymerase chain reaction
ST: sequence type
THA: THB agar
THB: Todd Hewitt broth
TNF: tumor necrosis factor
Zn: zinc
TPEN: Tetrakis-(2-Pyridylmethyl) ethylenediamine

## Declarations

### Data availability

The materials and data not presented in this manuscript are available from the corresponding author upon request.
