# Supplementary figures and images for: Laminin-binding protein of Streptococcus suis serotype 2 influences zinc acquisition and cytokine responses
Source: Vet Res. 2023 Jan 5;54:1. doi: 10.1186/s13567-022-01128-8 (PMC9817373; doi:10.1186/s13567-022-01128-8)

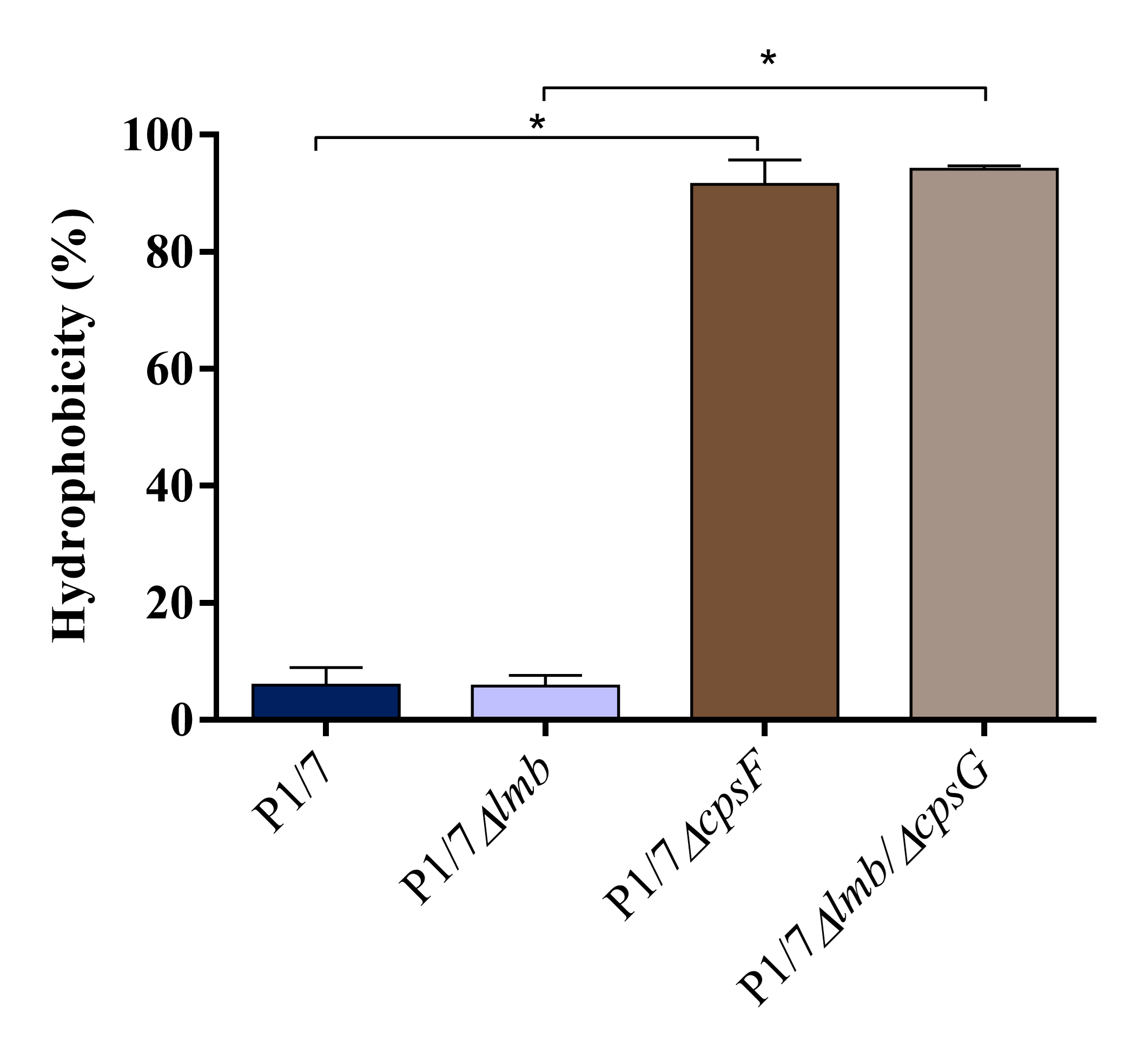

Supplement: Supplementary file 1 — Additional file 1: Absence of Lmb does not influence S. suis surface hydrophobicity. Surface hydrophobicity of of the wild-type P1/7 strain (dark blue), Δlmb (light blue), ΔcpsF (dark brown) and Δlmb/ΔcpsG (light brown) strains was determined using n-hexadecane. Data represent the mean ± SEM from at least three independent experiments. *Indicates a significant difference (p < 0.05). [file 13567_2022_1128_MOESM1_ESM.tif]
